# Leucine-Rich Glioma Inactivated-1 and Voltage-Gated Potassium Channel Autoimmune Encephalitis Associated with Ischemic Stroke: A Case Report

**DOI:** 10.3389/fneur.2016.00068

**Published:** 2016-05-09

**Authors:** Marisa McGinley, Sarkis Morales-Vidal, Sean Ruland

**Affiliations:** ^1^Department of Neurology, Loyola University Medical Center, Maywood, IL, USA; ^2^Department of Neurology, Florida Hospital Tampa, Tampa, FL, USA

**Keywords:** leucine-rich glioma inactivated-1, voltage-gated potassium channel, autoimmune encephalitis, limbic encephalitis, ischemic stroke

## Abstract

Autoimmune encephalitis is associated with a wide variety of antibodies and clinical presentations. Voltage-gated potassium channel (VGKC) antibodies are a cause of autoimmune non-paraneoplastic encephalitis characterized by memory impairment, psychiatric symptoms, and seizures. We present a case of VGKC encephalitis likely preceding an ischemic stroke. Reports of autoimmune encephalitis associated with ischemic stroke are rare. Several hypotheses linking these two disease processes are proposed.

## Introduction

Autoimmune encephalitis is associated with a wide variety of antibodies and clinical presentations. Voltage-gated potassium channel (VGKC) antibodies are a cause of autoimmune encephalitis that is not typically associated with an underlying tumor. The disorder is characterized by memory impairment, psychiatric symptoms, and seizures (1). More recent research has identified several antibodies responsible for the VGKC spectrum of diseases. Currently, there are three main categories: leucine-rich glioma inactivated-1 (LGI1), contactin-associated protein (Caspr2), and VGKC with unknown antigen. LGI1 is associated with the classic limbic encephalitis presentation, whereas anti-Caspr2 can present with encephalitis, peripheral nerve hyperexcitability, or Morvan syndrome (2, 3). To our knowledge, there is only one case report of VGKC autoimmune encephalitis associated with ischemic stroke (4). In this report, we describe a case of LGI1- and VGKC-positive autoimmune encephalitis that preceded a stroke in a young patient with no significant vascular risk factors and discuss the possible relationship. This case report was approved by our local IRB and exempt from informed consent because all identifying patient information was removed.

## Background

**Figure 1:**
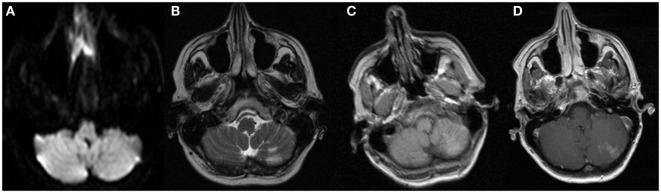
**MRI sequences from admission to the OSH 2 days prior to admission at our hospital**. These images demonstrate the cerebellar subacute infarction with no restricted diffusion, but contrast enhancement. These images remained unchanged 2 days later on admission to our hospital. **(A)** DWI, **(B)** T2, **(C)** T1 precontrast, and **(D)** T1 postcontrast.

**Figure 2:**
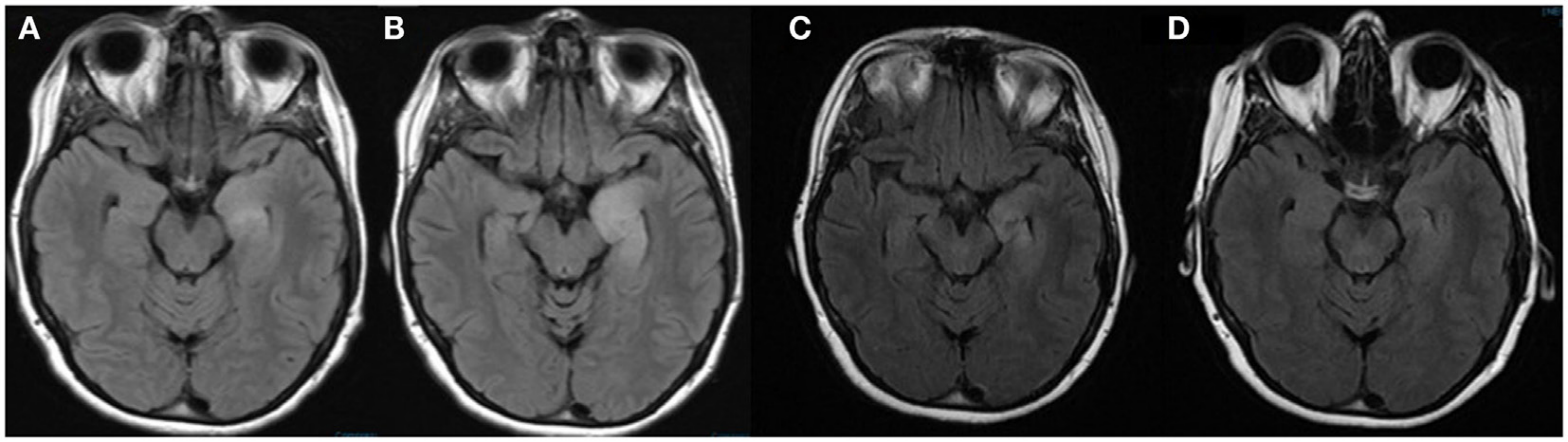
**MRI brain FLAIR sequences performed on admission to our hospital (A), 7 days after admission (B), 1 month after discharge (C), and 4 months after discharge (D)**. These images demonstrate the evolution of the left mesial temporal lobe lesion during admission and improvement 1 month and 4 months after discharge.

A 45-year-old woman with a history of depression and recently diagnosed with hypertension presented to our emergency department after several months of progressive neurological symptoms. Her symptoms initially began several months before. She described flashing lights in her peripheral vision occurring several times daily and was evaluated at another hospital with a brain MRI with and without contrast and a 24-h EEG, which were both reportedly unremarkable. Her visual symptoms were spontaneously resolved but then reoccurred 2 months later. Additionally, she developed right facial twitching and abnormal movements of her right arm. These episodes gradually increased in frequency until they were occurring multiple times daily. She was evaluated again at another hospital and empirically placed on levetiracetam 500 mg twice daily for presumed seizures. Another 24-h EEG at this time was unremarkable. Her episodes became more frequent and the intensity of the right upper extremity jerking worsened. On the second admission, she had a brain MRI with and without contrast that demonstrated several new abnormalities (Figures 1 and 2), another unremarkable prolonged EEG, and a lumbar puncture. Her cerebral spinal fluid demonstrated a protein of 76 (normal range 12–60 mg/dL), glucose of 62 (normal range 40–70 mg/dL), 1 WBC (normal range 0–10/mm^3^), 2 red blood cells (RBCs) (normal = 0), and a negative HSV PCR. Valproic acid was added, and she was discharged home.

**Figure 3:**
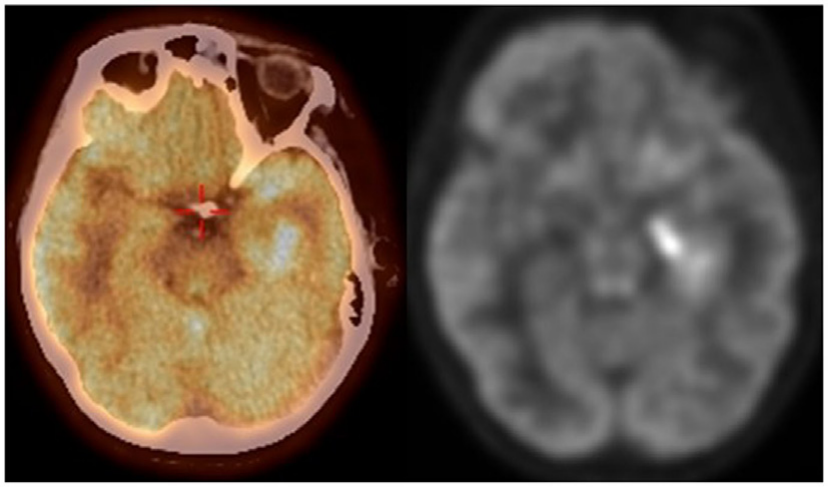
**FDG PET CT scan demonstrating increased FDG uptake in the medial left temporal lobe corresponding to the area of increased FLAIR signal seen on MRI brain**.

Two days following discharge, she presented to our emergency department with increasing frequency of right facial twitching and right upper extremity jerking despite compliance with the prescribed levetiracetam and valproic acid. Her husband also reported short-term memory impairment. Her neurological exam was significant for right hand finger abduction and wrist extension weakness along with diffuse hyperreflexia. There was no nystagmus, dysmetria, or gait ataxia. A repeat brain MRI with and without gadolinium contrast demonstrated a wedge-shaped lesion in the left cerebellar hemisphere with enhancement and without restricted diffusion. The appearance was consistent with a subacute ischemic infarction that was unchanged from the images obtained the week before (Figure 1). There was also T2 hyperintensity on fluid attenuation inversion recovery (FLAIR) sequences in the left hippocampus, which was unchanged when compared to the images obtained 1 week before (Figure 2). Continuous video EEG captured several episodes of impaired consciousness with abnormal right face and arm movements, but no electrographic seizures were seen. She underwent investigations for both the stroke and left hippocampal lesion. Transthoracic and transesophageal echocardiograms were both unremarkable. No arrhythmias were seen on telemetry during her entire 21-day hospitalization. MRA head and neck and CT angiography of the head were unremarkable. A hypercoagulable work-up, including lupus anticoagulant, cardiolipin antibody, beta2-glycoprotein, lipoprotein (a), antithrombin III, protein C, protein S, serum homocysteine, fibrinogen, and activated protein C resistance, was unremarkable. She underwent two lumbar punctures (LP) during her admission at our hospital. The first LP was performed 8 days after her previous LP at another hospital and had a protein of 25 (normal range 15–45 mg/dL), glucose of 53 (normal range 45–75 mg/dL), 0 WBC (normal range 0–8/UL), and 0 RBC (normal 0/UL). The second LP at our hospital was performed 18 days after the initial LP at the previous hospital and was significant for protein of 78, glucose of 71, 1 WBC, and 11 RBCs. Her clinical picture and left temporal lesion on imaging were consistent with limbic encephalitis. CT of the chest, abdomen, and pelvis was unremarkable for malignancy. A whole body PET scan also demonstrated no evidence of systemic malignancy, but a hypermetabolic area in the left mesial temporal lobe in the region of the hyperintensity seen on T2 FLAIR was present (Figure 3). Ma2, CRMP5, amphiphysin, anti Yo, Zic4, anti-Hu, and NMDA antibodies in CSF were negative. However, CSF anti-LGI1 was present and serum VGKC, which was >650 pmol/L (negative <450, borderline 450–650, and positive >650 pmol/L), was present. She was empirically treated with IV methylprednisolone1000 mg daily for 5 days and subsequently given 5 days of IVIG 400 mg/kg/day after the antibody results were reported. Maintenance treatment with mycophenolate mofetil 250 mg twice daily and an oral prednisone taper (50 mg × 3 days, then 40 mg × 3 days, and then 30-mg maintenance) was prescribed. Antiepileptic medications were discontinued. The episodes of adventitial movement and altered sensorium were consistent with faciobrachial dystonic seizures well described to occur in association with LGI1 autoimmune encephalitis. These seizures often do not have an EEG correlation and do not respond to antiepileptic drugs. They decreased in frequency throughout the hospital course. The right hand weakness improved and was resolved by the time of discharge. Her cognitive impairment improved, but she had residual difficulty with word recall and short-term memory at the time of discharge. Her only identifiable vascular risk factor, recently diagnosed hypertension, was treated with lisinopril and metoprolol. She was discharged on aspirin 81 mg daily. At her outpatient follow-up 1 month and 4 months after discharge, she had had no subsequent ischemic events and her memory had progressively improved. Repeat imaging 1 month and 4 months post-hospitalization showed near complete resolution of the mesial temporal lobe lesion (Figure 2).

## Discussion

This case illustrates a presentation of a premenopausal woman with classic findings of LGI1 subtype of VGKC antibody-associated autoimmune encephalitis and a recent cryptogenic ischemic stroke. To our knowledge, there has been only one report of a patient, published by Simal et al., with autoimmune encephalitis associated with stroke (4). He was a man in his sixth decade with hypertension, hyperlipidemia, and coronary artery disease, who was initially admitted with a middle cerebral artery distribution ischemic infarction. He developed symptoms of autoimmune encephalitis during his stroke admission, and it seemed clear that the stroke preceded the autoimmune encephalitis. The authors hypothesized that the stroke leads to disruption of the blood brain barrier allowing for easier access of the autoantibodies to the CNS. In our case, it is not clear which disease process occurred first. Given that the stroke appeared subacute on imaging after months of symptoms consistent with autoimmune encephalitis, it is reasonably surmised that the autoimmune encephalitis preceded the stroke. If this is the case, could autoimmune encephalitis be a risk factor for ischemic stroke? We propose several potential explanations.

One possibility is that the systemic inflammation during an autoimmune encephalitis leads to a prothrombotic state. Previous reports have described depressed levels of circulating antithrombotic-activated protein C and elevated levels of C4b-binding protein. These were primarily young patients with stroke and recent infection (5). Autoimmune encephalitis could also be associated with or upregulate another antibody that mediates vascular damage. This has been described in the case of antiendothelial cell antibodies, which are associated with several connective tissue disorders, including systemic lupus erythematous, granulomatosis with polyangiitis, and Susac’s syndrome (5–7). Finally, the stroke could have occurred independently and potentiated the autoimmune encephalitis. The patient’s symptoms were indolent for several months, but when she was presented with more florid cognitive decline and increased seizures, the ischemic stroke was first visualized. Blood–brain barrier injury from the stroke could have allowed easier access of antibodies to the CNS, as suggested by Simal et al., resulting in worsening of her condition.

Ultimately, the outcomes for these patients are promising. Current literature indicates that syndromes associated with an antibody against a neural surface antigen as opposed to an intracellular or paraneoplastic antigen often respond better to immunotherapy (3). The first line therapies for VGKC spectrum disorders include steroids, intravenous immunoglobulins, and plasma exchange. These treatment modalities can be used alone or in combination (8). If the patient is refractory to these treatments, rituximab or cyclophosphamide has been suggested due to their effectiveness in other forms of limbic encephalitis (3). A recent systematic review analyzed specific outcomes for clinical recovery and MRI findings (9). Neuropsychiatric symptoms were often subjectively better, but objective outcomes were more variable. MRI findings often have significant improvement, but residual focal atrophy has also been described (8). The most significant improvements are reported in patients with a non-paraneoplastic etiology, affective predominant symptoms, classical radiological findings, and large decreases in antibody titers (9).

## Conclusion

In conclusion, this case is an example of LGI1 autoimmune encephalitis associated with ischemic stroke that raises questions regarding possible relationships between these entities.

## Author Contributions

MM and SR both were involved with the care of this patient during admission and with the initial write up of the case. SM-V also cared for this patient during the admissions as an outpatient and helped with editing and review of the paper.

## Conflict of Interest Statement

The authors declare that the research was conducted in the absence of any commercial or financial relationships that could be construed as a potential conflict of interest.
